# THE ETHICS OF AN OPT-OUT DEFAULT IN TOBACCO TREATMENT

**DOI:** 10.1111/add.12820

**Published:** 2015-02-11

**Authors:** Richard E Ashcroft

**Affiliations:** School of Law, Queen Mary University of LondonLondon, UK

**Keywords:** Behaviour change, medical ethics, nudges, public health, tobacco, tobacco treatment

In their paper 1, Richter & Ellenberg advance an argument that a key way to improve public health is to reduce the number of smokers by increasing the uptake of tobacco cessation (and, one might add, harm reduction) programmes by current smokers. They note that in many health systems the default position is that patients seeking clinical care are only offered tobacco treatment if, in the opinion of the clinician, they are either expressing a wish to quit smoking or otherwise give signs of ‘readiness to quit’. They argue further that this creates a barrier to treatment, which can be removed by mandating clinicians to offer tobacco treatment to all patients who smoke without assessing ‘readiness to quit’, leaving the decision to the patients as to whether or not they take up this offer of treatment. The theory here is that some patients who might otherwise not have been considered ‘ready to quit’ by their clinicians will accept the offer of treatment, and that some of those will complete treatment successfully. Moreover, it is assumed that no (or minimally few) patients who would accept treatment under the current default will reject it under the new default. While the authors mention the possibility that the simple offer of treatment, by communicating implicitly that the treatment will be beneficial to the patient, will actively influence some patients to agree, they do not rely on this influence as the mechanism of action underlying the intervention. They simply suggest that offering treatment to more people will probably lead to more people accepting the offer, with net beneficial consequences.

The strongest part of their practical argument is the claim that requiring clinicians to assess the patient's readiness to quit before offering treatment is inefficient and unreliable in identifying which patients will accept treatment and may complete it. What this boils down to, when we strip away the fashionable language of ‘nudges’, is the proposal that clinicians should offer tobacco treatment to any patient who needs it, and that the test of need is simply that the patient is a tobacco user. It is the patient's behaviour which is the clinical sign, not their psychological state (readiness or otherwise to quit). This seems reasonable. The authors present some suggestive evidence which indicates that offering treatment to all smokers is effective, and it does not put off patients from accepting or completing treatment.

The ethical status of the proposed shift to an ‘opt-out’ default is more complicated. One detail is that the default being changed here is the default for the clinician, not the patient. In any medical treatment, the patient is offered treatment in line with the clinician's judgement of what is in their best interests, and the patient refuses or accepts. In this sense, all medical treatment has an opt-out default from the patient's point of view. Thus, Richter & Ellenberg's intervention is really targeted at clinicians’ behaviour. The question then arises as to whether they have shown whether there is evidence for the efficacy of interventions to change clinicians’ behaviour; they have not. Some clinicians will offer treatment, some will not, as before. The intervention here is, in a sense, to call ‘offering all patients treatment’ a ‘nudge’ or ‘default change’ will be more or less influential on clinicians’ behaviour.

Another detail concerns the impact of this intervention on the clinician–patient relationship. It is already debated whether treating lawful, albeit harmful, behaviours as clinical disorders is acceptable to patients. Similarly, there is room for debate over whether there is a distinction between treating illness and actively promoting health and wellbeing, and whether, if such a distinction holds, there is a difference between a moral imperative to promote health and wellbeing in the same way that there is a clinical obligation to treat illness. Pure ethics aside, at least some patients will resent the offer of tobacco treatment when their primary presenting complaint is something (they say as) unrelated—for instance, arthritis or depression. One way to put the point is this: assessing the ‘readiness to quit’ might be understood as ‘getting to know the patient’ and establishing a clinical relationship which has the quality of care and respect. Simply requiring clinicians to default to an offer of treatment as a matter of standard practice without taking note of the importance of this relationship might prove to be offensive to some patients and counterproductive in more.

In conclusion, while Richter & Ellenberg have a sensible proposal, we need a better understanding of whether it works in practice, and what impact it has on clinician–patient relationships, before we can agree that an opt-out for the offer of treatment is mandatory best practice.
